# Renal disease progression in autosomal dominant polycystic kidney disease

**DOI:** 10.1007/s10157-012-0611-9

**Published:** 2012-04-21

**Authors:** Eiji Higashihara, Shigeo Horie, Satoru Muto, Toshio Mochizuki, Saori Nishio, Kikuo Nutahara

**Affiliations:** 1 Department of Urology, Kyorin University School of Medicine, 6-20-2 Shinkawa, Mitaka, Tokyo 181-8611 Japan; 2 Department of Urology, Teikyo University School of Medicine, Tokyo, Japan; 3 Department of Internal Medicine, Hokkaido University Graduate School of Medicine, Sapporo, Japan

**Keywords:** Autosomal dominant polycystic kidney disease, Glomerular filtration rate, Kidney volume, Kidney function, Kidney failure

## Abstract

**Background:**

Autosomal dominant polycystic kidney disease is a lifelong progressive disorder. However, how age, blood pressure, and stage of chronic kidney disease (CKD) affect the rate of kidney function deterioration is not clearly understood.

**Methods:**

In this long-term observational case study up to 13.9 years (median observation period for slope was 3.3 years), serum creatinine was serially measured in 255 mostly adult patients. The glomerular filtration rate was estimated (eGFR) using a modified Modification of Diet in Renal Disease Study method. The total kidney volume (TKV) has been measured in 86 patients at one center since 2006.

**Results:**

As age increased, eGFR declined significantly (*P* < 0.0001), but the annual rate of decline of eGFR did not correlate with age or initially measured eGFR. In patients with CKD stage 1, eGFR declined at a rate which was not significantly different from other advanced CKD stages. Hypertensive patients had lower eGFR and larger TKV than normotensive patients at a young adult age. The slopes of regression lines of eGFR and TKV in relation to age were not different between high and normal blood pressure groups.

**Conclusion:**

The declining rate of eGFR was relatively constant and did not correlate with age or eGFR after adolescence. eGFR was already low in young adult patients with hypertension. As age increased after adolescence, eGFR declined and TKV increased similarly between normal and high blood pressure groups. eGFR starts to decline in patients with normal eGFR, suggesting that the decline starts earlier than previously thought.

**Electronic supplementary material:**

The online version of this article (doi:10.1007/s10157-012-0611-9) contains supplementary material, which is available to authorized users.

## Introduction

Progressive deterioration of renal function and enlargement of renal cysts are two hallmarks of autosomal dominant polycystic kidney disease (ADPKD). It is widely recognized that during the renal compensation period, renal function decreases slowly but subsequently decreases at a relatively faster rate [1, 2]. In a three-year CRISP study [3], the rate of change in iothalamate clearance was faster in the older age group (>30 years) than in the younger group, but the difference was not statistically significant (*P* = 0.2). Even if the glomerular filtration rate (GFR) is maintained near normal at a young adult age, ADPKD patients already have decreased effective renal plasma flow and an increased filtration fraction [4]. A recent study revealed that occurrence of glomerular hyperfiltration in ADPKD children is associated with a significantly faster decline in renal function and higher rate of kidney enlargement over time [5]. As a result of more severe progression of ADPKD children with glomerular hyperfiltration, GFR is already lower than normal at around adolescent. Long-term longitudinal studies delineating renal disease progression are limited.

Currently, potential therapeutic interventions are being developed for ADPKD [6–11]. The potentially effective compounds examined so far seem not to reverse already decreased renal function or decrease already enlarged kidney volume but to mitigate progressive deterioration or enlargement [6–8, 11]. The mammalian target of rapamycin inhibitors, appeared to retard the growth of kidneys but not to slow functional deterioration in patients with ADPKD who have stage 2 or 3 chronic kidney disease (CKD) [8, 10]. Tolvaptan, a V2-specific vasopressin receptor antagonist, slowed cyst growth progression in ADPKD patients compared to historical controls [11]. In animal experiments, it was suggested that intervention with a V2-specific vasopressin receptor antagonist should be early in ADPKD [18].

It is not known how the declining rate differs between CKD stage 1 patients through to CKD stage 3 patients with ADPKD. It is important to delineate the characteristics of the natural course of disease progression in ADPKD when therapeutic intervention becomes feasible.

## Materials and methods

Two hundred and fifty-five patients with ADPKD participated in an observation study at Kyorin University, Teikyo University and Hokkaido University from 1995 to 2009. The patients fulfilled Ravine’s diagnostic criteria. The study was an observational case study measuring serum creatinine at least once a year and monitoring blood pressure. Serum creatinine was measured enzymatically. The estimated glomerular filtration rate (eGFR, ml/min/1.73 m^2^) was calculated using the following formula: eGFR (male) = 194 × Cr^−1.094^ × Age^−0.287^, and eGFR (female) = eGFR (male) × 0.739. This equation is a Japanese coefficient for the modified Isotope Dilution Mass Spectrometry-Modification of Diet in Renal disease (IDMS-MDRD) Study [12]. The slopes of the reciprocal of serum creatinine concentration (1/Cr) were also examined. The slopes of eGFR (ml/min/1.73 m^2^/year) and 1/Cr (dl/mg/year) were calculated when creatinine was measured for at least two points with an interval longer than 12 months. Slopes were calculated using linear regression analysis in each patient. The staging of kidney function is based on the K/DOQI Clinical Practice Guidelines on CKD [13].

Since 2006, total kidney volume (TKV) has been measured at Kyorin University Hospital in routine clinical practice by high-resolution magnetic resonance imaging using volumetric measurement of cross-sectional imaging, as described in the report from the Consortium for Radiologic Imaging Studies in Polycystic Kidney Disease (CRISP) [3, 14, 15]. Gadolinium enhancement was not used for safety concerns. The TKV slope is calculated using linear regression analysis and is expressed as the yearly change of TKV (ml/year).

In the present study, hypertension is defined as high blood pressure requiring the use of anti-hypertensive agents. In the three hospitals where the study was conducted, blood pressure >130/85 was commonly treated by renin−antiotensin system blockers to achieve the target blood pressure.

For evaluation of the relationship between eGFR and TKV, data were analyzed when eGFR and TKV were measured within 1 month. As eGFR and TKV were measured several times in one patient, initial measurement data were used to examine age-related changes of eGFR and TKV. This protocol was approved by an institutional review board, and the study was conducted in accordance with the guidelines of the Declaration of Helsinki. All participants gave written informed consent to use their clinical data for medical research.

### Statistical analyses

Analyses were performed with Microsoft Excel 2003, SAS 9.1 for Windows. Parametric variables are expressed as the mean ± standard deviation. Two-sided *P* < 0.05 was considered to indicate statistical significance. *P* values for differences between CKD stages were obtained using ANOVA or the Kruskal–Wallis test. Correlations between two variables were examined by linear regression analysis. The correlation coefficient (*r*) was obtained by the Spearman rank-order correlation coefficient. The relations of two linear regression lines between normotensive and hypertensive groups were compared by *F* test. Student’s *t* test was used to calculate the *P* value between two age groups.

## Results

Pertinent data in groups according to the measured parameters are shown in Table 1. eGFR was measured in 255 patients and eGFR slope was calculated in 196 patients whose eGFR was measured more than twice and more than 12 months apart. TKV was measured in 86 patients and the TKV slope was calculated in 46 patients.

**Table 1:** Pertinent data on kidney function and volume according to the measured parameters

Data	Groups according to the measured parameters			
	eGFR^a^	eGFR slope^c^	TKV^b^	TKV slope^c^
Patient number	255	196	86	46
Male/female	99/156	80/116	34/52	18/28
Age (years)	44.9 ± 14.2	46.0 ± 13.8	47.0 ± 14.2	45.1 ± 14.5
Mean observation period (years)	3.3 ± 3.1	4.2 ± 3.0	0.8 ± 0.8	1.4 ± 0.5
Median observation period (years)	2.5	3.3	0.8	1.3
AntiHTN Tx/no antiHTN Tx^a^	184/71	153/43	67/19	35/11
eGFR (ml/min/1.73 m^2^)^b^	62.4 ± 37.0	61.2 ± 33.1	63.4 ± 32.1	71.5 ± 29.4
eGFR slope^c^ (ml/min/1.73 m^2^/year)	−	−3.4 ± 4.9	–	–
eGFR slope/initial eGFR (%/year)	–	−7.4 ± 8.9	–	–
1/Cr slope (dl/mg/year)	–	−0.05 ± 0.08	–	–
TKV (ml)	–	–	1839.4 ± 1329.2	1675.0 ± 944.4
TKV slope^c^ (ml/year)	–	–	–	86.8 ± 161.6
TKV slope/initial TKV (%/year)	–	–	–	5.6 ± 8.8
Log TKV slope^d^ (log ml/year)	–	–	–	0.02 ± 0.04
Log TKV slope/initial log TKV (%/year)	–	–	–	0.7 ± 1.2
Observation period of TKV slope (years)	–	–	–	1.4 ± 0.5

*TKV* total kidney volume

^a^AntiHTN Tx/no antiHTN Tx: patient number with and without anti-hypertensive treatment. HTN Tx is indicated for BP higher than 130/85 mmHg

^b^eGFR is estimated GFR measured the first time

^c^Slope is the annual change of eGFR or TKV

^d^Log TKV slope is log (TKV2/TKV1)/year

**Fig. 1:**
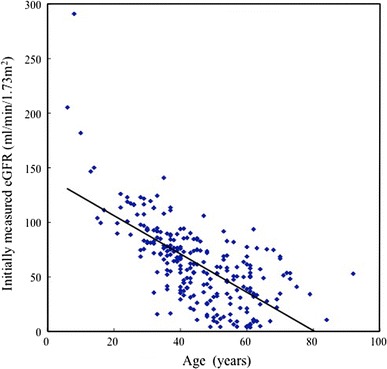
Initially measured eGFR distribution in relation to age (*n* = 255). *y* = −1.757*x* + 141.28, *r* = −0.6871, *P* < 0.0001

Initially measured eGFR in relation to age is shown in Fig. 1. eGFR decreased statistically significantly as age increased (*P* < 0.0001).

**Fig. 2:**
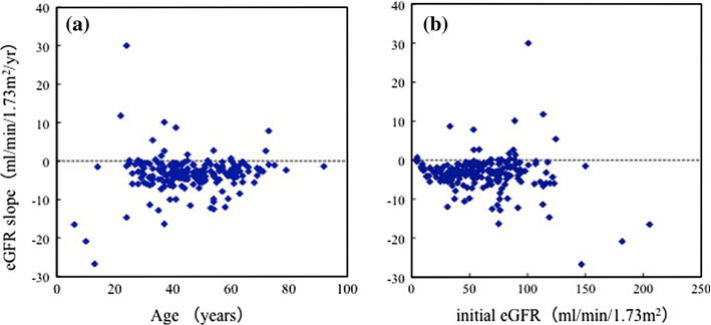
Relationship of eGFR slope to age (**a**) and initial eGFR (**b**) (*n* = 196). **a** Spearman’s rank correlation coefficient (*r*) = 0.0728, *P* = 0.3094. **b** Spearman’s rank correlation coefficient (*r*) = −0.0412, *P* = 0.5654. No significant relationship is seen between eGFR slope and age, or between eGFR slope and initially measured eGFR. Mean observation time of eGFR was 4.2 ± 3.0 years

The change in eGFR per year (eGFR slope) was plotted against age and initially measured eGFR in 196 patients (Fig. 2a, b). The regression lines were not statistically significant. The result suggests that eGFR slope does not relate to age or initially measured eGFR.

In Table 2, 196 patients are grouped according to the CKD stage [13] depending on the initially measured eGFR. The advancement of CKD stages significantly related to increased age (*P* < 0.0001). Slopes of eGFR and 1/Cr were not statistically different among CKD stages, and even younger patients with relatively preserved kidney function in stage 1 had similar slopes of eGFR and 1/Cr to patients in advanced stages. The percent ratio of the decline in eGFR and 1/Cr in relation to the initially measured values progressively increased as the CKD stage advanced (*P* < 0.0001).

**Table 2:** Age, eGFR slope and 1/Cr slope in relation to the CKD stages of initially measured eGFR

	CKD stages according to initially measured eGFR^a^ (ml/min/1.73 m^2^)				*P* value
	Stage 1 ≥90	Stage 2 89–60	Stage 3 59–30	Stage 4 + 5^b^ ≤29	
Initial eGFR (ml/min/1.73 m^2^)	113.8 ± 25.9	75.1 ± 7.9	45.0 ± 8.8	16.3 ± 8.0	–
Patient number	32	62	71	31	–
Age (years)	29.9 ± 11.4	42.4 ± 10.2	52.4 ± 12.1	55.0 ± 8.4	<0.0001
eGFR slope^c^ (ml/min/1.73 m^2^/year)	−4.2 ± 9.5	−3.5 ± 4.1	−3.1 ± 3.3	−2.8 ± 1.7	0.6775
eGFR slope/initial eGFR × 100 (%/year)	−3.2 ± 8.0	−4.8 ± 5.4	−7.5 ± 8.5	−16.4 ± 10.3	<0.0001
1/Cr slope^d^ (dl/mg/year)	−0.04 ± 0.13	−0.05 ± 0.07	−0.06 ± 0.07	−0.05 ± 0.03	0.8982
1/Cr slope/initial 1/Cr × 100 (%/year)	−2.2 ± 7.4	−4.0 ± 5.1	−6.7 ± 8.1	−15.1 ± 9.6	<0.0001

Data are presented as the mean ± SD. *P* values are calculated by ANOVA

^a^Patients were staged according to the National Kidney Foundation Disease Outcomes Quality Initiative guidelines

^b^ESRD (dialysis and transplantation) is not included in stage 4 and 5 groups

^c^eGFR slope is the annual change of estimated GFR

^d^1/Cr slope is the annual change of 1/Cr

**Fig. 3:**
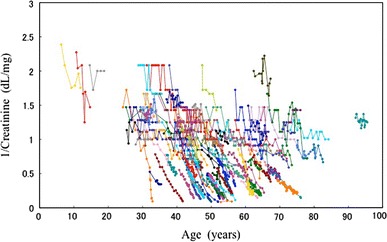
1/Cr is plotted against age in 106 patients who had been followed up for more than 3 years. 1/Cr declines in most patients at an individually variable rate. Pattern of decline appears not to be age-dependent

**Fig. 4:**
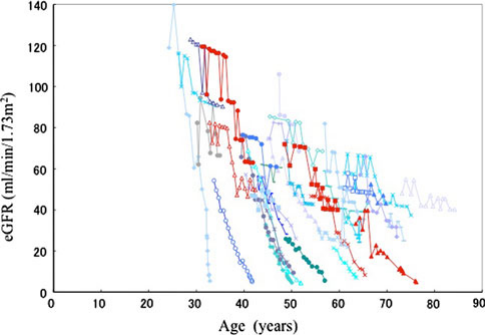
eGFR changes in patients followed for more than 5 years (*n* = 36). In 5 patients shown by a *red line*, the declining curve changed from moderate to rapid during follow up. The change points varied in relation to age or eGFR level. Other patients are shown in *blue* for easy identification

1/Cr was plotted against age in 106 patients who had been followed for more than 3 years (Fig. 3). In the supplementary figure, the plot of 1/Cr versus age is illustrated in all 255 patients. 1/Cr declined to a greater or lesser extent every year with a relatively constant decline rate for each patient at considerable variance among individuals. Neither figure shows that 1/Cr remains stable at a younger age than at an older age. For more detailed examination of the compensatory period of GFR, eGFR is plotted against age in 36 patients who had been followed up for more than 5 years (Fig. 4). Similar to 1/Cr, eGFR declined in each patient. In five patients shown by red lines, the declining curve changed from moderate to rapid during follow-up. The change points did not show any age or eGFR level dependency.

The effects of age on the eGFR and TKV slopes are examined in Table 3. Forty-six patients whose TKV slopes were measured were divided into younger or older age groups for comparison purposes. Between the two groups, the difference in eGFR was statistically significant but differences in the eGFR slope, 1/Cr slope, TKV or TKV slope were not significant.

**Table 3:** Comparison of the slopes of eGFR and TKV between the two age groups

	Younger group	Older group	*P* value
Age group (years)	13–41	42–75	
Mean age (years)	34 ± 6.4	57 ± 10.5	
Male/female	11/12	7/16	
eGFR (ml/min/1.73 m^2^)	87.0 ± 29.5	55.9 ± 19.7	<0.0001
eGFR slope (ml/min/1.73 m^2^/year)	−4.6 ± 7.3	−2.1 ± 3.1	0.1540
eGFR slope/initial eGFR (%/year)	−4.2 ± 9.2	−4.4 ± 7.6	0.9640
1/Cr slope (dl/mg/year)	−0.06 ± 0.10	−0.03 ± 0.06	0.3876
1/Cr slope/initial 1/Cr × 100 (%/year)	−3.0 ± 8.1	−3.8 ± 7.1	0.7535
TKV (ml)	1509.3 ± 874.3	1840.8 ± 1001.2	0.2381
TKV slope (ml/year)	110.2 ± 207.5	63.5 ± 96.0	0.3326
TKV slope/initial TKV (%/year)	7.6 ± 10.3	3.6 ± 6.6	0.1215
Log TKV slope (log ml/year)	0.03 ± 0.04	0.01 ± 0.03	0.1877
Log TKV slope/initial log TKV (%/year)	0.9 ± 1.4	0.4 ± 1.0	0.1580

Forty-six patients whose TKV slopes were measured were divided into younger and older age groups for comparison. Data are the mean ± SD. *P* values were calculated by Student’s *t* test

**Fig. 5:**
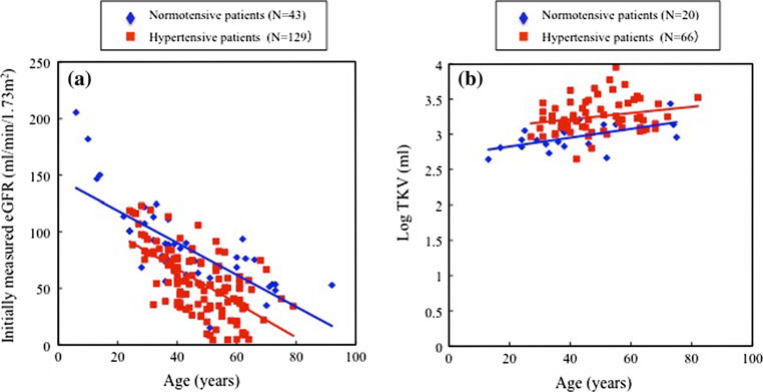
**a** Initially measured eGFRs are plotted against age in normotensive (*blue*) and hypertensive (*red*) patients. Regression analysis for normal blood pressure group: *y* = 151.08 − 1.546*x* (where *y* = eGFR and *x* = age, *r* = −0.7791, *P* < 0.0001, *n* = 70) and that for hypertensive group: *y* = 132.30 − 1.666*x* (*r* = −0.6587, *P* < 0.0001, *n* = 158). **b** The relationship between age and log-transformed TKV in normotensive (*blue*) and hypertensive (*red*) patients. Regression analysis for normal blood pressure group; *y* = 2.7003 + 0.006275*x* (where *y* = log TKV and *x* = age, *r* = 0.57859, *P* = 0.0075, *n* = 20) and that for hypertensive group; *y* = 3.0339 + 0.004452*x* (*r* = 0.23144, *P* = 0.0615, *n* = 66). In both **a** and **b**, the regression lines for normotensive and hypertensive patients were not considered to be identical, with different *y*-intercepts, since there was a significant difference (*P* < 0.01, *F* test) in the *y*-intercept of the two regression lines under the null hypothesis that the *y*-intercept of two lines was identical. There was no significant difference (*P* = 0.6061 in **a** or *P* = 0.6079 in **b**, *F* test) in the slope of the two lines under the null hypothesis that the slope of the two lines was identical

The initially measured eGFRs and log-transformed TKV are plotted against age in normotensive and hypertensive patients in Fig. 5a, b, respectively. In both figures, the regression lines for normotensive and hypertensive patients were not considered to be identical, with different *y*-intercepts, since there was a significant difference (*P* < 0.01, *F* test) in the *y*-intercept of the two regression lines under the null hypothesis that the *y*-intercept of the two lines was equal. There was no significant difference (*P* = 0.6061 in Fig. 5a or *P* = 0.6079 in Fig. 5b, *F* test) in the slope of the two lines under the null hypothesis that the slope of the two lines was equal.

Table 4 shows that in young adult patients aged <36 years, eGFR was lower and TKV was larger in the hypertensive group than in the normal blood pressure group.

**Table 4:** Comparison of eGFR and TKV between normal and high blood pressure groups in young adults (≤35 years)

	Normotensive group	Hypertensive group	*P* value
*N*	36	27	
Initial BP^a^			
Systolic (mmHg)	117.9 ± 15.1	148.1 ± 14.2	<0.0001
Diastolic (mmHg)	68.5 ± 6.9	85.9 ± 13.7	0.0001
Post-Tx BP^b^			
Systolic (mmHg)	115.8 ± 14.4	128.4 ± 12.9	0.0030
Diastolic (mmHg)	70.5 ± 11.6	78.4 ± 6.5	0.0066
eGFR (ml/min/1.73 m^2^)	113.6 ± 42.5	86.6 ± 24.2	0.0044
*N*	10	12	
TKV (ml)	826.3 ± 319.2	1713.2 ± 675.6	0.0011

Data are the mean ± SD. *P* values were calculated by Student’s *t* test

^a^Initial BP is blood pressure without anti-hypertensive treatment in hypertensive group and blood pressure at initial visit in normotensive group

^b^Post-Tx BP is blood pressure at the study time. In hypertensive group, all patients were receiving antihypertensive medication

## Discussion

ADPKD is the most common hereditary kidney disease. The disease is characterized by the formation of numerous kidney cysts and their development, leading to kidney enlargement and failure, reaching end-stage renal failure in up to about 50% by age 70 [16].

Polycystic kidney disease animal model studies suggested that earlier intervention resulted in more effective prevention of disease progression [17, 18]. The potential candidates clinically examined so far seem to attenuate progression but not to reverse progressed renal disease [6–8, 11]. Thus, it is a crucial issue when to start treatment intervention.

The present study confirmed that renal function decreased progressively as a function of age [1, 3, 16, 19, 20]. In 196 patients with a mean age >30 years, the mean eGFR slope was −3.4 ± 4.9 ml/min/1.73 m^2^/year. In 46 patients with mean TKV >1500 ml, the TKV slope was 86.8 ± 161.6 ml/year (5.6 ± 8.8%/year) (Table 1). The present data of eGFR and TKV slopes are compatible with previous findings [3, 10]. The slopes of GFR (measured by iothalamate clearance) and TKV were analyzed according to TKV and age groups in the CRISP study [3]. Analysis of variance revealed that the slopes of GFR differed among subgroups with different initial TKV (*P* = 0.005), whereas the slopes of GFR did not differ significantly among subgroups with different initial ages (*P* = 0.20); there was no significant interaction between TKV and age (*P* = 0.95) [3]. In the present study, the eGFR slope was less in the older group than younger group (Table 3), but the difference was not statistically significant (*P* = 0.154). In addition, there was no significant relationship between age and eGFR slope (Fig. 2a). Both the present and CRISP study [3] suggest that the eGFR slope is not significantly affected by age, at least after adolescence.

The MDRD equation for estimating GFR is widely used [8–10] but its accuracy was recently reported to be 83% in ADPKD patients [21]. Renal function changes are qualitatively reflected by the 1/Cr slope in individual subjects, because individual body muscle volume and hydration status are relatively stable in most patients, at least for relatively short periods of a few years. In the present study, the 1/Cr slope was analyzed in addition to the eGFR slope and the results were qualitatively similar in both analyses (Tables 2, 3; Figs. 3, 4).

In 5 of 36 patients followed for more than 5 years, renal disease progression accelerated during observation (Fig. 4). This acceleration did not seem to be related to age or eGFR level, but presumably to individually different causes, including infection, hematuria, obstruction by urolithiasis or other events. If the acceleration of renal disease progression is due to the end of the renal compensation mechanism, the terminal points of the compensation mechanism might be heterogeneous among ADPKD patients.

In relatively younger adult (29.9 ± 11.4 years) patients whose renal function was retained (CKD stage 1 in Table 2), the eGFR slope was already negative. In the majority of patients with initially measured eGFR >90 ml/min/1.73 m^2^, the eGFR slope was negative, as shown in Fig. 2b. These results suggest that the renal compensation mechanism might terminate in the second decade of life in most patients with ADPKD.

A recent study which examined the detailed renal functions of young ADPKD patients showed abnormal kidney function even in the younger generation [4]. In a quartile of the younger age group (27 ± 5 years) in that study, GFR decreased but was statistically not different from that of the normal healthy controls. Even in these younger age group patients, effective renal plasma flow sharply decreased. Patients with CKD stage 1 (Table 2) in the present study correspond to quartile 1 group patients in that study [4], because age (29.9 ± 11.4 vs 27 ± 5 years) and eGFR (113.8 ± 25.9 ml/min/1.73 m^2^) in the present study and GFR measured by iothalamate clearance (117 ± 32 ml/min) were not statistically different. The present study shows a negative eGFR slope and the study [4] showed decreased renal plasma flow in similar younger adult patients who maintained apparently normal GFR.

Initially measured eGFR in relation to age in hypertensive patients was lower than that in normotensive patients, and the present results indicated that differences in eGFR between the two groups had already occurred before age 36 (Fig. 5a; Table 4). Hypertensive children with ADPKD were reported to be at particular risk for increases in renal volume and decreased renal function as compared with children with normal blood pressure. Renal function was already decreased by age 20, at least in hypertensive children [20]. The important finding in the present study is that declining rates of eGFR and increasing rates of TKV are not significantly different between normal blood pressure and high blood pressure patients after around 20 years. This phenomenon might or might not be due to anti-hypertensive treatment. The results of previous [20] and present studies suggest that renal functional deterioration starts far earlier than 20 years of age, especially in hypertensive ADPKD patients.

The potential limitations of this study include retrospective analysis, use of eGFR and 1/Cr, as well as an ethnically homogenous patient population in Japan, and hence it may not be applicable to other ethnicities.

## Conclusions

In conclusion, eGFR starts to decline in young adult patients with apparently normal eGFR. After adolescence, the declining rate of eGFR is relatively constant and does not relate to age or GFR. Hypertensive patients had lower eGFR and larger TKV than normotensive patients at young adult age. After adolescence, eGFR declined at a similar rate between normotensive and hypertensive groups. A long-term longitudinal study starting in childhood is necessary to more thoroughly understand the characteristics of disease progression in ADPKD.

## Electronic supplementary material

Below is the link to the electronic supplementary material.

1/Creatinine is plotted against age in all 255 patients (JPEG 87 kb)
