# Supplementary figures and images for: Renal disease progression in autosomal dominant polycystic kidney disease
Source: Clin Exp Nephrol. 2012 Apr 21;16(4):622–8. doi: 10.1007/s10157-012-0611-9 (PMC3416980; doi:10.1007/s10157-012-0611-9)

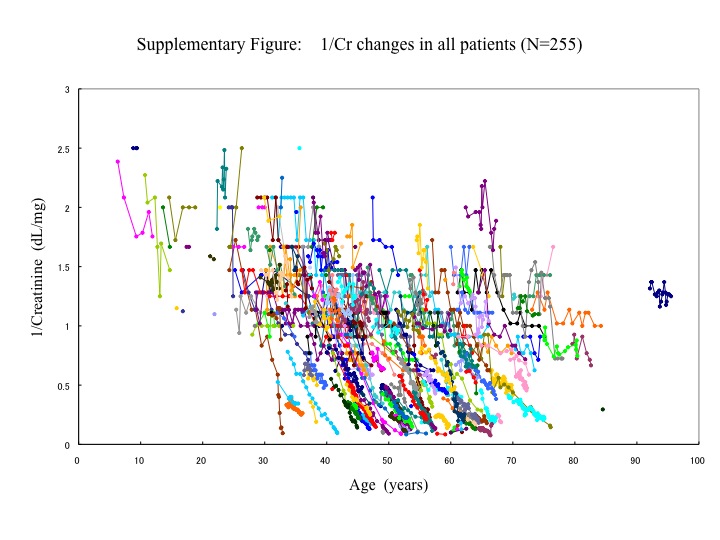

Supplement: Supplementary file 1 — 1/Creatinine is plotted against age in all 255 patients (JPEG 87 kb) [file 10157_2012_611_MOESM1_ESM.jpg]
